# Osteogenesis Imperfecta: Current and Prospective Therapies

**DOI:** 10.3390/biom11101493

**Published:** 2021-10-10

**Authors:** Malwina Botor, Agnieszka Fus-Kujawa, Marta Uroczynska, Karolina L. Stepien, Anna Galicka, Katarzyna Gawron, Aleksander L. Sieron

**Affiliations:** 1 Department of Molecular Biology and Genetics, Faculty of Medical Sciences in Katowice, Medical University of Silesia, Medykow 18, 40-752 Katowice, Poland; afus@sum.edu.pl (A.F.-K.); marta.uroczynska@sum.edu.pl (M.U.); kbugaj@sum.edu.pl (K.L.S.); kgawron@sum.edu.pl (K.G.); alsieron@sum.edu.pl (A.L.S.); 2 Department of Medical Chemistry, Medical University of Bialystok, Mickiewicza 2A, 15-222 Bialystok, Poland; angajko@umb.edu.pl

**Keywords:** osteogenesis imperfecta, treatment, mesenchymal stem cells, gene therapy, iPSCs

## Abstract

Osteogenesis Imperfecta (OI) is a group of connective tissue disorders with a broad range of phenotypes characterized primarily by bone fragility. The prevalence of OI ranges from about 1:15,000 to 1:20,000 births. Five types of the disease are commonly distinguished, ranging from a mild (type I) to a lethal one (type II). Types III and IV are severe forms allowing survival after the neonatal period, while type V is characterized by a mild to moderate phenotype with calcification of interosseous membranes. In most cases, there is a reduction in the production of normal type I collagen (col I) or the synthesis of abnormal collagen as a result of mutations in col I genes. Moreover, mutations in genes involved in col I synthesis and processing as well as in osteoblast differentiation have been reported. The currently available treatments try to prevent fractures, control symptoms and increase bone mass. Commonly used medications in OI treatment are bisphosphonates, Denosumab, synthetic parathyroid hormone and growth hormone for children therapy. The main disadvantages of these therapies are their relatively weak effectiveness, lack of effects in some patients or cytotoxic side effects. Experimental approaches, particularly those based on stem cell transplantation and genetic engineering, seem to be promising to improve the therapeutic effects of OI.

## 1. Introduction

Osteogenesis Imperfecta (OI) (types I–V, MIM 166200, 166210, 259420, 166220, and 610967) includes a diverse group of heritable connective tissue disorders with a broad range of phenotypes characterized primarily by bone fragility. The first scientific description of this disease appeared in 1788, and since then the nomenclature and classification started to evolve. Over time, new clinical observations, such as blue sclera, hearing impairment, deafness, hypermobility, and hyperlaxity of the joints have been associated with OI. Additionally, in some cases, dental abnormalities have been reported. The development of molecular biology and radiological methods in the late 1970s allowed establishing an OI classification based on clinical symptoms. Four types of disease have been distinguished: type I, characterized by dominantly inherited OI with blue sclera and dentinogenesis imperfecta, type II, including lethal perinatal OI with radiographically crumpled femora and beaded ribs, type III, progressively deforming OI, and type IV that includes a dominantly inherited variant with normal sclera [1]. Although a defect in the collagen gene has been indicated for many years as the main cause of OI, a different mode of inheritance, i.e., autosomal dominant—identified for OI types I and IV—and autosomal recessive—for types II and III—indicated genetic heterogeneity of this disease and the possibility that defects of other genes can induce the development of OI [2]. Accordingly, over time, the Sillence’s classification of OI phenotypes has shown some limitations. First, it describes a gradual spectrum of severity including secondary features, such as blue sclera, hearing impairment, and teeth abnormalities, which are not credible classification criteria due to their high variability. Second, this classification did not include patients whose OI diagnosis was based on skeletal and bone histological features and who did not reveal any defects in the primary sequence of collagen. Furthermore, most of the included cases were characterized by the autosomal recessive mode of inheritance. Further confusion in OI classification resulted from the identification of defects in new genes as the disease etiologic factor and the fact that new variants of OI defined by their aetiology showed a significant clinical overlap with “classical” variants characterized previously by Sillence [2]. Therefore, during recent years, OI classification has been revised and modified several times. The most recent classification of genetic skeletal disorders defined in 2019 distinguishes five types of OI, including the types I to IV from the original Sillence’s classification and an additional type V, described as OI with calcification of interosseous membranes and/or hypertrophic callus [3,4]. In most cases, OI is inherited in an autosomal dominant mode, but autosomal-recessive and X-chromosome-linked variants of the disease have been also reported. It has been established that the main cause of OI are mutations in col type I genes [5]. Recent research has identified mutations in a couple of new genes, mostly involved in col I synthesis and processing, as well as in genes encoding some transcription factors and signalling proteins involved in osteoblast/osteoclast differentiation [6,7,8,9,10,11,12,13,14,15]. Table 1 presents the most recent classification, including the mode of inheritance, affected genes, and clinical characteristics of OI phenotypes.

In this review, we discuss current treatment options and provide insights into upcoming OI therapies mostly based on genetic engineering and stem cell transplantation.

## 2. Review

### 2.1. Current Treatment of OI

Currently, available treatment options for OI include prevention of bones fractures, control of symptoms, and increase of bones mass. The treatment modes of OI include both non-surgical and surgical procedures. The non-surgical approach includes physical therapy, braces, and splints being used to prevent deformity and promote support and protection, as well as the use of medications. Surgical intervention may be used to deal with local pathologies, such as bone fractures, bowing of bones, or scoliosis. To counteract the overall systemic effects, medications have to be prescribed.

The most commonly used drugs in the treatment of OI are bisphosphonates (BPs). These compounds were introduced as OI treatment to increase bone mass density and prevent fractures. Although the main target of BPs are osteoclasts, they also interact with osteoblasts and osteocytes. BPs inhibit osteoclasts in the basic multicellular unit turn-over cycle where new remodelling sites are created, while pre-existing ones are filled with osteoblasts, improving the ratio of bone formation to bone resorption and resulting in increased bone mass density [16,17]. At present, two kinds of BPs are under use, non-nitrogen-containing BPs, which cause apoptosis of osteoclasts by forming analogues of ATP, and nitrogen-containing BPs, which do not have such effect. Moreover, BPs without nitrogen have higher affinity for hydroxyl apatite crystals, especially in metabolically active trabecular bone. Currently, BPs with even higher affinity to hydroxyapatite crystals, such as Zoledronate and Pamidronate, are the most commonly used medications [18].

The safety of intravenous bisphosphonates (IV BPs) has been studied for the treatment of osteoporosis and low bone mineral density in children with spinal muscular atrophy (SMA) [19]. Small acute side effects were observed, including fever (after Pamidronate/zoledronic acid injection), diarrhoea after the first dose and dysautonomic storm following subsequent zoledronic acid infusion. Furthermore, one week after dosing, hypocalcaemia and hypophosphatemia were observed in 5% and 73% of the patients, respectively. In another study, patients treated with oral BPs revealed intolerable gastrointestinal side effects or progressive dysphagia [20]. Apart from the side effects reported, BPs fail to improve the connectivity of the bone tissue and are most effective only during the first year of their administration [21,22]. Another disadvantage of using BPs is their relatively long half-life, which may even last for several years when attached to bones. Moreover, BPs are not effective in all OI patients [23]. Thus, new drugs with shorter half-life and different mechanisms of action need to be introduced into clinical practice.

Denosumab is a monoclonal antibody (IgG_2_) against receptor activator of nuclear factor kappa-B ligand (RANKL), which inhibits osteoclast formation without binding to bone [24]. The advantage of Denosumab is a relatively short degradation period, which lasts for around three to four months, avoiding the long-term accumulation side effects of BPs [25]. This compound studied in patients with OI types I, III, IV and VI not responsive to BPs has shown promising benefits with relatively high safeness [25,26]. Moreover, the research from a large, phase III clinical FREEDOM trail in postmenopausal women with osteoporosis after 10 years of treatment with Denosumab revealed sustained elevation of bone mineral density, with only low rates of adverse events and a low fracture incidence [27,28]. Otherwise, the negative effects reported for Denosumab include rebound effects after stopping the treatment, hypercalcaemia, and hypercalciuria during the treatment [24]. These reports indicate that further studies on this compound are still required.

**Figure 1:**
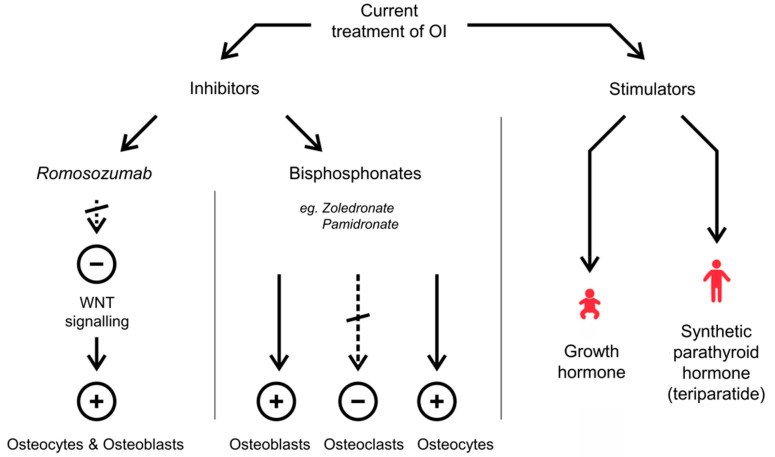
Schematic overview of currently used medications to treat OI. The use of some inhibitors allows for increased bone mass by inhibiting the osteoclast cycle while inducing osteoblasts and osteocytes, e.g., *Zoledronate* and *Pamidronate*, the most commonly used BPs with high affinity for hydroxyapatite crystals. In contrast, Romosozumab, which exerts its effect by inhibiting the Wingless-type MMTV integration site family, member 1 (WNT) signalling involved in osteocytes–osteoblasts stimulation, and stimulators, such as growth hormone and teriparatide, induce bone formation instead of inhibiting osteoclast function. Drug names are indicated in italics.

Another class of drugs used in the therapy of OI is directed to stimulate bone formation instead of inhibiting osteoclast function. It has been reported that growth hormone positively affects bone strength in children with growth hormone deficiency; it was thought that it might also work in children with severe OI. However, growth hormone showed only limited improvement in bone mass density compared to BPs and, currently, it is not used as a therapeutic in children with OI [29]. It has been shown that in adults with OI, synthetic parathyroid hormone (teriparatide), which is also used in postmenopausal osteoporosis treatment, leads to an increase in bone mass density [30,31,32]. Teriparatide was not tested in children due to the increased risk of osteosarcoma reported in animal studies [33]. Another drug with a positive effect in adults suffering from OI is Romosozumab. It is a glycoprotein that inhibits Wingless-type MMTV integration site family, member 1 (WNT) signalling involved in osteocytes—osteoblasts stimulation. After OI patients’ treatment with Romosozumab, increased bone mass density and concomitant increase in blood markers characteristic for bone formation have been demonstrated [34]. Similar effects were reported in patients with postmenopausal osteoporosis treated with Romosozumab, where an increase in bone mineral density was observed in the lumbar spine, femoral neck and total hip bone [35]. However, the Active–Controlled Fracture Study in Postmenopausal Women with Osteoporosis at High Risk (ARCH) showed increased rates of adjudicated serious cardiovascular adverse events in the Romosozumab group compared with the Alendronate group [36]. Furthermore, Lv et al. in their meta-analysis indicated that Romosozumab might increase the risk of complex cardiovascular outcomes, referred to as four-point major adverse cardiovascular event (4 P-MACE), including myocardial infarction, stroke, heart failure and death among patients with primary osteoporosis. Due to divergent information on the side effects of this drug, further studies with longer term follow-up are needed [37]. An overview of the currently used treatments for OI is presented in Figure 1.

### 2.2. Experimental Strategies for OI Therapy

#### 2.2.1. Anti-TGF-β Antibodies

The development of new treatment modes and therapy schemas for OI predominantly depends on a better understanding of the genetic background and molecular mechanisms of this disease. It is clear now that transforming growth factor-beta (TGF-β) exerts an effect on both osteoblasts and osteoclasts [38]. Inhibition of TGF-β is thought to silence the over-activation of TGF-β signalling, which is involved in the regulation of bone mass and fractures in OI, and the use of anti-TGF-β antibodies allows bone mass to increase and therefore also improves bone strength [28,39]. These results seem promising in OI treatment but also indicate the need for further investigations on the use of this antibody in humans and on Losartan as an angiotensin II-receptor agent with anti-TGF-β properties [40,41]. Another drug, Fresolimumab, a GC1008 antibody that inhibits TGF-β, previously used to treat cancer patients [42], is currently in clinical trials to verify its safety and efficacy in the treatment of OI in adult patients [39,43]. Moreover, Rice et al. reported improved inhibition of TGF-β-regulated genes expression in response to Fresolimumab in a study on systemic sclerosis patients [44]. The drug requires further studies but seems promising for the effective treatment of OI in the future.

#### 2.2.2. Stem Cells Transplantation

Mesenchymal stem cells (MSCs) are very promising for the treatment of bone diseases because of their ability to differentiate into cells such as osteoblasts, osteocytes and chondrocytes [45].

Results of stem cell treatments depend mostly on the type of transplant, i.e., whole bone marrow, MSCs, human foetal mesenchymal stem cells (hfMSCs), transplantation technique, and the age of the animals employed in the studies [46]. Some stem cell therapies have been already applied to humans. Increase in growth and mineral content and decrease in fractures rates were reported in OI patients with bone marrow transplanted from HLA-matched siblings [47]. These effects, however, were not stable over time, and additional treatment with isolated bone marrow/mesenchymal stem cells (BMSCs) was needed. However, the second trial resulted in a sustained positive outcome of the previous transplantation. Other cases of prenatal and postnatal transplantation of hfMSCs in patients with severe OI were reported by Götherström et al. Transplantations resulted in improved growth and decreased number of fractures. Moreover, no alloreactivity to donor hfMSCs or possible toxic reactions to the procedure were observed [48]. Other clinical trials with HLA-matched family members were conducted [49]. Successful application of stem cell therapy in paediatric patients, both prenatally (in utero) and postnatally, has been also reported in other studies [28,39]. Prenatal treatment seems particularly promising, since it would enable the formation of healthy bones and a normal skeleton at the earliest possible age. Despite these attractive results, treatment based on transplantation is still considered ambiguous, because of a small number of treated patients and largely insufficient empirical evidence. To use these methods in routine clinical practice, further trials are needed.

#### 2.2.3. Methods Based on Genetic Engineering and Somatic Cells Reprogramming into iPSCs

Another approach for OI treatment may involve the correction of defects in genes. There are currently several ways to modify collagen mutant transcripts. The recent approach consists in converting the severe type of OI based on structural defects in col I protein into a less severe quantitative form by silencing or inactivating the mutant gene, leading to allele suppression and haploinsufficiency [50]. Different methodological approaches can be used to silence collagen mutant transcripts, for example, the use of antisense oligodeoxyribonucleotides (ODNs), short interfering RNA (siRNA), and hammerhead ribozymes. These strategies were tested in various studies involving in vitro, ex vivo and, to a lesser extent, animal OI models. Results of siRNA silencing of the *COL1A1* gene in vitro and ex vivo have shown a reduction of the amount of mutant RNA of 50% and of the mutant protein of 40% [28,51]. Although the results are interesting, these techniques are still in the experimental stage. There are several aspects that need to be addressed, such as the specific design of the silencing molecules, the creation of carrier agents to deliver them into target cells, and technical details regarding the application. The diverse locations of mutations responsible for OI complicate the process of gene editing and make the design of universal silencing molecules very difficult. Moreover, with this approach, potential risks of immune response and genotoxicity should also be taken into consideration [52]. Additionally, the duration of the positive effects of the therapy are unknown; therefore, clinical trials are still required [28].

The use of induced pluripotent stem cells (iPSCs) represents another interesting approach to OI therapy, recently attracting the attention of researchers worldwide. It can be achieved by employing mouse embryonic or adult fibroblasts reprogrammed genetically to a pluripotent state due to transfection with Yamanaka factors, i.e., Oct3/4, Sox2, Klf4 and c-Myc (OSKM), or Thomson factors, i.e., Oct3/4, Sox2, Lin28a and Nanog (OSLN), using Sendai virus. Patient-specific iPSCs are a valuable tool for genetic disease treatment and are useful in drug screening [53]. Besides, iPSCs have a significant advantage compared to embryonic stem cells (ESCs), because in the first case, mature somatic cells from patients who suffer from genetically defined diseases are used [54,55,56]. Moreover, iPSCs have a great potential to differentiate into cells of each of the three germ layers, i.e., ectoderm, mesoderm, and endoderm [57]. Although viral transfection is the most common procedure and is much more effective in comparison with currently available non-viral methodologies, the invention of an equally sufficient but safer transfection method is of great importance [54]. So far, many cell types, such as hepatocytes (endoderm origin), circulating T cells (mesoderm) and keratinocytes (ectoderm) have been reprogrammed into iPSCs. However, the most commonly used cells are skin fibroblasts, mostly because of their easy accessibility [54,55,57]. iPSCs have been used for the first time in a clinical trial (first phase) in 2014 in Japan for retinitis pigmentosa treatment [58]. Additionally, iPSCs generated in a patient-specific manner have therapeutic potential in autologous transplants for treatment of retinitis pigmentosa, haemophilia A, severe combined immunodeficiency (SCID), myotonic dystrophy and Sandhoff disease [59]. Therefore, personalized iPSCs constitute an invaluable cell type for use in replacement therapy. Until now, iPSCs have been obtained from peripheral blood mononuclear cells (PBMCs) and used for OI type I treatment [53]. The ability of blood cells reprogramming is a novel perspective for clinical practice, as blood collection is a standard test. Additionally, HLA matching has a crucial role in the limitation of alloimmune responses [59].

**Figure 2:**
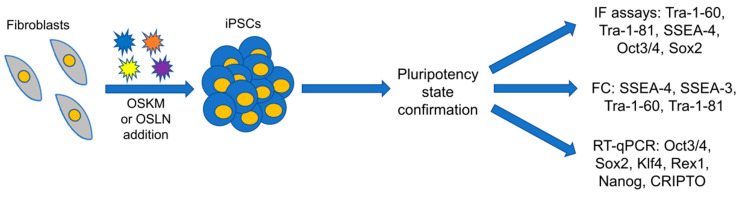
Schematic presentation of somatic cells reprogramming into iPSCs and pluripotency analysis. The generation of iPSCs from fibroblasts and the pluripotency state confirmation are presented. The most commonly used markers of pluripotency are shown. OSKM, Yamanaka factors (Oct3/4, Sox2, Klf4, c-Myc); OSLN, Thomson factors (Oct3/4, Sox2, Lin28a, Nanog); iPSCs, induced pluripotent stem cells.

Another way to reprogram somatic cells is the direct introduction of transcriptional factors (Yamanaka or Thomson factors) into cells in the protein form. Despite the efficiency of this method being lower compared to virus-based reprogramming, some improvements may be applied. The hydrophobicity of the plasma membrane and the large size of the introduced proteins may be overcome by fusion of the proteins with a Cell Penetrating Peptide (CPP) such as trans-activator of transcription (TAT) or 11-Arginine residues (11R) [56,60]. This may be a promising alternative to viral vectors in the reprogramming procedure, making this approach integration-free. In turn, nanoparticles such as synthetic polymers may be used as carriers of biological material, whereas a combination of a recombinant protein fused with CPP and nanoparticles allows enhancing the effect of CPP. Importantly, the lack of star polymers’ cytotoxicity has been already confirmed, enabling their safe use in gene therapy and patient-specific therapies [61]. The state of pluripotency needs also to be confirmed by the expression of pluripotency genes or by specific antibodies (Figure 2).

CRISPR–Cas9 is currently a well-established genome editing tool widely used in various experimental models, including human clinical trials. In vivo delivery of Cas9-guide RNA complexes to repair abnormal genes has been successfully developed in murine models of autosomal dominantly inherited diseases [62]. In the case of OI, the CRISPR–Cas9 system was successfully used in mice, iPSCs lines and blood cells-derived iPSCs [63,64,65,66]. According to Peng et al., iPSCs corrected by the gene editing technology acquire the potential to repair pathological lesions and completely cure the disease [54]. Moreover, it has been demonstrated that the modification of iPSCs harbouring mutations using CRISPR–Cas9 gene editing tools is of great significance for individual therapies of genetic vascular diseases [54]. The combination of iPSCs generation with methods such as CRISPR-based genome editing may improve iPSCs-based gene therapy. It is, therefore, difficult to exclude that future applications of genome editing tools will cure at least the milder forms of OI.

#### 2.2.4. Counteraction of ER Stress and UPR

A recently proposed attractive target of OI therapy is ER stress caused by intracellular retention of mutant collagen in osteoblasts and fibroblasts [67,68,69,70]. The use of the FDA-approved chemical chaperone 4-phenylbutyrate (4-PBA), which additionally exhibits histone deacetylase inhibitor activity, ameliorated cell homeostasis in fibroblasts from dominant and recessive OI patients and in the Chihuahua zebrafish model of the classical dominant OI [67,68,69]. Administration of 4-PBA to this zebrafish resulted in an increase in collagen secretion, a decrease in the size of ER cisternae and an improvement of the skeletal phenotype, i.e., bone mineralization in larvae and skeletal deformities in adult fish [69]. Treatment with 4-PBA has been also shown to alleviate cellular stress by restoring the normal size of ER cisternae, normalizing the expression of the apoptosis marker caspase-3 and decreasing the activation of the unfolded protein response (UPR) in Brtl and Amish mouse models. In addition, the drug promotes collagen secretion and its incorporation into the ECM [70]. Treatment with 4-PBA of fibroblasts from dominant OI patients with α1(I) mutations facilitated collagen folding and secretion, decreased the expression of eukaryotic translation initiation factor 2 alpha kinase 3 (PERK) and reduced the activation of apoptosis [67]. Moreover, as a histone deacetylase inhibitor, 4-PBA increased the expression of the autophagic *Atg5* gene and stimulated cell autophagy. The beneficial effect of this chaperone was also demonstrated for recessive OI with mutations in the 3-hydroxylation complex (CRTAP, P3H1 and PPIB). As in dominant OI, the reduction in the accumulation of misfolded collagen, the restoration of ER cisternae size, and decreased apoptosis were found after drug administration [68]. Furthermore, this chaperone exerted a stimulatory effect on autophagy. Interestingly, in the in vitro study employing iPSCs, 4-PBA was shown to promote osteogenic gene expression and mineralization, while reducing abnormal collagen synthesis and UPR markers [71].

**Figure 3:**
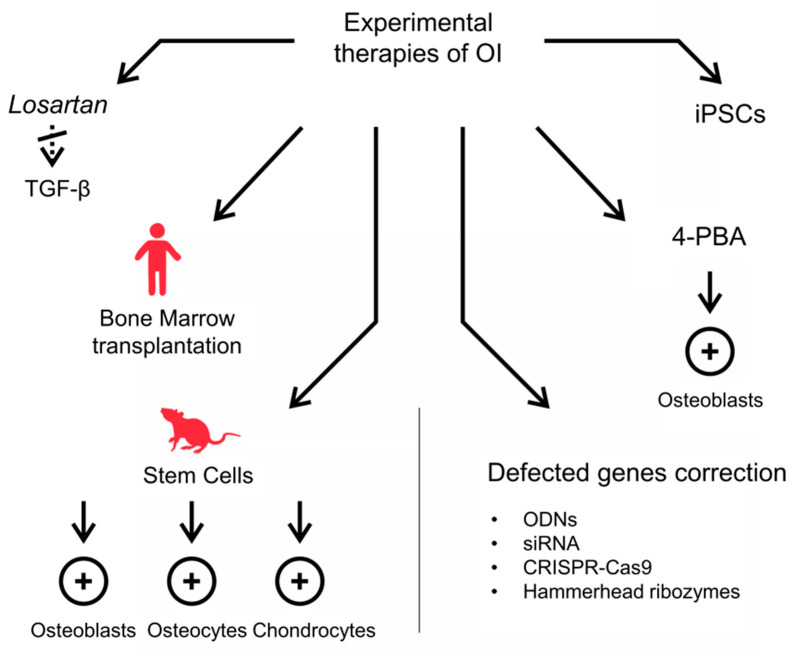
Summary of experimental strategies for OI therapy tested in in vitro, ex vivo, animal models and clinical trials. The promising strategies include (i) the use of Losartan, an angiotensin II-receptor agent with anti-TGF-β properties; (ii) mesenchymal stem cells transplantation as the source of osteoblasts, osteocytes, and chondrocytes; (iii) correction of defective genes, including ODNs, antisense oligodeoxyribonucleotides; siRNA, short interfering RNA; CRISPR–Cas9 and hammerhead ribozymes; (iv) restoring cell homeostasis to improve the severity of the OI phenotype using chemical chaperones, e.g., 4-PBA, 4-phenylbutyrate; (v) iPSCs, induced pluripotent stem cells use.

Restoring cell homeostasis to improve the severity of the OI phenotype may be a more promising therapeutic strategy than correction of the structural abnormalities associated with the secretion of mutant collagen into the ECM, and 4-PBA is a potential common factor in the treatment of recessive and classic forms of OI due to intracellular collagen accumulation. A summary of the current experimental strategies for OI therapy is presented in Figure 3.

## 3. Conclusions

In recent decades, OI has moved from a disease with unknown aetiology to a disorder with a precisely described and mapped genetic background. The currently available treatment options of OI try to prevent fractures, control symptoms and increase bone mass. The most common medications used in OI treatment are bisphosphonates, Denosumab, synthetic parathyroid hormone and growth hormone for children therapy. The main disadvantages of these therapies are their relatively weak effectiveness, the lack of effects or the appearance of treatment resistance in some patients or cytotoxic side effects. Promising strategies for the future treatment of OI and other genetic bone diseases are based on stem cell transplantation, genetic engineering, and molecular chaperones usage. However, most of these approaches are still in the experimental stage. Consequently, further investigations are needed to confirm their therapeutic benefits in OI.

## Figures and Tables

**Table 1:** Classification of OI phenotypes.

Type of OI	Inheritance Mode	Mutated Gene(s)	O(MIM) Number (Gene)	Clinical Characteristics
Osteogenesis imperfecta type I	AD	*COL1A1*	120150	Mild, non-deforming, with normal stature, increased bone fragility, blue-grey sclerae, hearing loss [5]
		*COL1A2*	120160	
Osteogenesis imperfecta type II	AD	*COL1A1*	120150	Lethal in the perinatal period [5]
		*COL1A2*	120160	
	AR	*CRTAP*	605497	Severe to lethal [6,7,8]
		*P3H1*	610339	
Osteogenesis imperfecta type III	AD	*COL1A1*	120150	Progressively deforming [5]
		*COL1A2*	120160	
		*IFITM5*	614757	Mild to moderate [9]
	AR	*SERPINF1*	172860	Moderate to severe, scoliosis [10]
		*CRTAP*	605497	Severe to lethal [6,7,8]
		*P3H1*	610339	
		*SERPINH1*	600943	Severe to lethal [11]
	AR	*FKBP10*	607063	Broad-spectrum, includes OI, Bruck syndrome, Kuskokwim syndrome (joint contractures at birth, short stature) [12]
	AR	*BMP1*	112264	Severe; high bone mass [13]
		*WNT1*	164820	Moderately severe [14]
Osteogenesis imperfecta type IV	AD	*COL1A1*	120150	Moderately deforming [5]
		*COL1A2*	120160	
		*WNT1*	164820	Moderately severe [14]
		*IFITM5*	614757	Mild to moderate [9]
	AR	*FKBP10*	607063	Progressive, deforming [12]
		*SP7*	606633	Moderate [15]
Osteogenesis imperfecta type V	AD	*IFITM5*	614757	Mild to moderate (moderately deforming) with calcification of interosseous membranes and/or hypertrophic callus [9]

Abbreviations: O(MIM), Online Mendelian Inheritance in Man; AD, autosomal dominant; AR, autosomal recessive; *COL1A1*, collagen type I, α1 chain; *COL1A2*, collagen type I, α2 chain; *CRTAP*, cartilage-associated protein; *P3H1*, propyl 3-hydroxylase-1; *IFITM5*, interferon-induced transmembrane protein 5; *SERPINF1*, pigment epithelium-derived factor (PEDF); *SERPINH1*, serpin peptidase inhibitor, clade h, member 1 encoding heat shock protein 47 (HSP47); *FKBP10*, an FKBP-type peptidyl-prolyl cis-trans isomerase; *BMP1*, bone morphogenetic protein 1; *WNT1*, Wingless-type MMTV integration site family, member 1; *SP7*, a transcriptional factor, osterix (Osx).

## Data Availability

Data sharing is not applicable to this article, as no new data were created in this study.
